# The NSP6-L260F substitution in SARS-CoV-2 BQ.1.1 and XBB.1.16 lineages compensates for the reduced viral polymerase activity caused by mutations in NSP13 and NSP14

**DOI:** 10.1128/jvi.00656-25

**Published:** 2025-05-13

**Authors:** Yuri Furusawa, Kiyoko Iwatsuki-Horimoto, Seiya Yamayoshi, Yoshihiro Kawaoka

**Affiliations:** 1 The Research Center for Global Viral Diseases, National Center for Global Health and Medicine Research Institute 350198, Shinjuku, Tokyo, Japan; 2 Division of Virology, Institute of Medical Science, University of Tokyo 13143, Bunkyo, Tokyo, Japan; 3 The University of Tokyo Pandemic Preparedness, Infection and Advanced Research Center, Tokyo, Japan; 4 International Research Center for Infectious Diseases, Institute of Medical Science, University of Tokyo 13143, Bunkyo, Tokyo, Japan; 5 Department of Pathobiological Sciences, School of Veterinary Medicine, University of Wisconsin-Madison 5229, Madison, Wisconsin, USA; University of Kentucky College of Medicine, Lexington, Kentucky, USA

**Keywords:** SARS-CoV-2, COVID-19

## Abstract

Severe acute respiratory syndrome coronavirus 2 (SARS-CoV-2) Omicron variants emerged at the end of 2021, and their subvariants are still circulating worldwide. While changes in the S protein of these variants have been extensively studied, the roles of amino acid substitutions in non-structural proteins have not been fully revealed. In this study, we found that SARS-CoV-2 bearing the NSP6-L260F substitution emerged repeatedly when we generated several SARS-CoV-2 variants by reverse genetics or when we passaged SARS-CoV-2 isolated from clinical samples and that it was selected under cell culture conditions. Although this substitution has been detected in BQ.1.1 and XBB.1.16 that circulated in nature, its effect on viral properties is unclear. Here, we generated SARS-CoV-2 with or without the NSP6-L260F by reverse genetics and found that NSP6-L260F promotes virus replication *in vitro* and *in vivo* by increasing viral polymerase activity and enhancing virus pathogenicity in hamsters. We also identified disadvantageous substitutions, NSP13-M233I and NSP14-D222Y, that reduced BQ.1.1 and XBB.1.16 replication, respectively. These adverse effects were compensated for by NSP6-L260F. Our findings suggest the importance of NSP6-L260F for virus replication and pathogenicity and reveal part of the evolutionary process of Omicron variants.

**IMPORTANCE:**

Although the properties of severe acute respiratory syndrome coronavirus 2 (SARS-CoV-2) Omicron variants continue to change through the acquisition of various amino acid substitutions, the roles of the amino acid substitutions in the non-structural proteins have not been fully explored. In this study, we found that the NSP6-L260F substitution enhances viral polymerase activity and is important for viral replication and pathogenicity. In addition, we found that the NSP13-M233I substitution in the BQ.1.1 lineage and the NSP14-D222Y substitution in the XBB.1.16 lineage reduce viral polymerase activity, and this adverse effect is compensated for by the NSP6-L260F substitution. Our results provide insight into the evolutionary process of SARS-CoV-2.

## INTRODUCTION

Severe acute respiratory syndrome coronavirus 2 (SARS-CoV-2) was identified as the causative agent of COVID-19 at the end of 2019 (1, 2). At the end of 2021, Omicron variants emerged, and their subvariants are still circulating worldwide. Numerous amino acid substitutions have been shown to alter the antigenicity of the spike protein, and several amino acid substitutions have also been detected in the nonstructural proteins (NSPs). However, the roles of these NSP substitutions have not been revealed.

The 5′ terminal two-thirds of the SARS-CoV-2 genome contains open reading frames (ORFs) 1a and 1b. From these ORFs, 16 NSPs are translated and play roles during virus replication. Coronaviruses, including SARS-CoV-2, form a membrane-bound replication organelle composed of connectors and double-membrane vesicles (DMVs) in infected cells (3). DMVs are formed by NSP3 and NSP4. NSP6 is a transmembrane protein that is essential for DMV organization and the connection between DMVs and the endoplasmic reticulum (ER) (4, 5). NSP6 has been reported to alter virus pathogenicity and contribute to the attenuation of Omicron BA.1 (6, 7). Viral RNA synthesis occurs in DMVs, catalyzed by the RNA-dependent RNA polymerase (RdRp), which comprises NSP12, NSP7, and NSP8 (3). NSP13 and NSP14 interact with the RdRp to support RNA synthesis, functioning as an RNA helicase and exonuclease/N7-methyltransferase, respectively (3).

In this study, we found that SARS-CoV-2 bearing the NSP6-L260F substitution emerged repeatedly under cell culture conditions and explored the substitution by examining recombinant viruses possessing this amino acid substitution *in vitro* and *in vivo*. Our findings revealed how this substitution affects the characteristics of SARS-CoV-2 Omicron variants.

## RESULTS

### Recognition of the NSP6-L260F substitution

**Fig 1:**
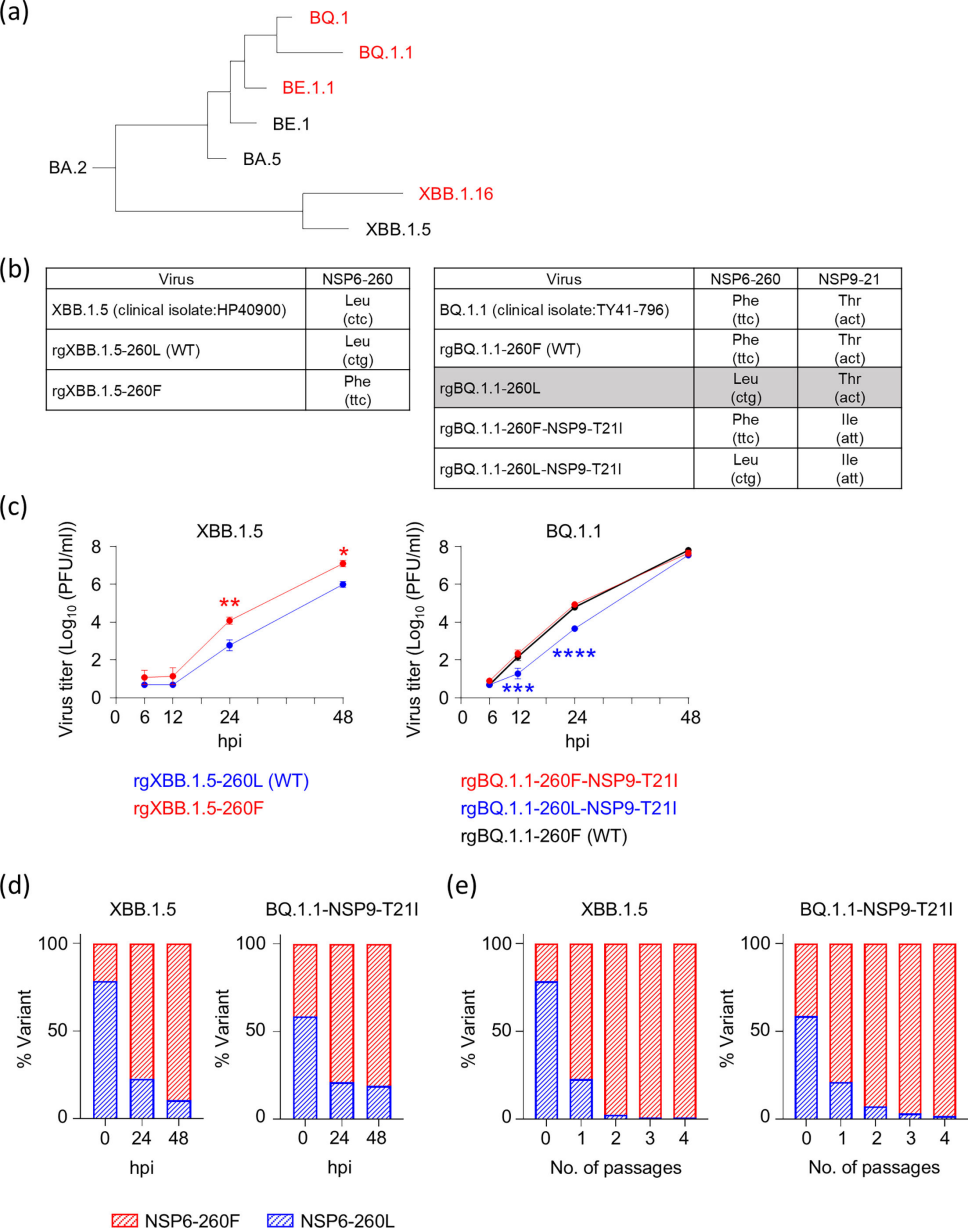
The effect of NSP6-L260F on the growth kinetics of SARS-CoV-2. (**a**) Phylogenetic tree of SARS-CoV-2 viruses. Lineages with the NSP6-L260F substitution are shown in red. (**b**) The amino acid and nucleotide sequences of the clinical isolate and viruses generated by reverse genetics are shown. We used a different codon for NSP6-260L compared to the clinical isolate to reduce the potential for reversion. As clonal rgBQ.1.1-260L (highlighted in gray) could not be rescued, we used BQ.1.1 viruses possessing NSP9-T21I in addition to the NSP6-L260F substitution for subsequent experiments. (**c**) VeroE6/TMPRSS2 cells were infected with each virus at a multiplicity of infection (MOI) of 0.0001. Virus titers at the indicated time points were determined by using plaque assays (*n* = 3, mean ± s.e.m.). (d and e) rgXBB.1.5-260L (WT) and rgXBB.1.5-260F or rgBQ.1.1-260F-NSP9-T21I and rgBQ.1.1-260L-NSP9-T21I were mixed at an equal ratio on the basis of their infectious titers, and the virus mixture was infected into VeroE6/TMPRSS2 cells grown on 24-well plates in triplicate at an MOI of 0.001. At the indicated time points or at each passage, the proportion of each virus was determined by deep sequencing analysis. Data were analyzed by using a two-way ANOVA with Dunnett’s multiple comparisons test. Statistical significance was calculated against the values in rgXBB.1.5-WT- or rgBQ.1.1-260F-NSP9-T21I-infected cells. ∗*P* < 0.05, ∗∗*P* < 0.01, ∗∗∗*P* < 0.001, and ∗∗∗∗*P* < 0.0001.

When we tried to rescue SARS-CoV-2 with reduced replicative capability by reverse genetics or passaged viruses isolated from clinical samples from BA.5 (8), BA.4.6 (9), or XBB.1.5-infected individuals, it took longer than usual for the cytopathic effect to spread throughout the cultured cells, resulting in the acquisition of the NSP6-L260F substitution in the propagated SARS-CoV-2. This observation implied that the NSP6-L260F substitution enhances virus replication. When we searched for this substitution in clinical isolates submitted to the GISAID database, we found that most of the BE.1.1, BQ.1, BQ.1.1, and XBB.1.16 lineages possess it (Fig. 1a). The BE.1.1 (BA.5.3.1.1.1) lineage emerged at the beginning of 2022, and its descendants, the BQ.1 and BQ.1.1 lineages, had rapidly spread to several countries by the end of 2022 (10). In contrast, the XBB.1.16 lineage evolved from the XBB.1.5 lineage (11) and outcompeted other variants in India in early 2023 (11, 12). Therefore, NSP6-L260F may be important for virus spread in humans, even though it is observed in limited lineages.

### NSP6-260F increases virus replication *in vitro*

To evaluate the effect of NSP6-L260F on virus replication, we generated rgXBB.1.5-260L (WT) and rgXBB.1.5 with NSP6-L260F (rgXBB.1.5-260F) by reverse genetics. For NSP6-260L, we used a different codon from the clinical isolate to avoid reversion (Fig. 1b). Similarly, we tried to generate rgBQ.1.1-260F (WT) and rgBQ.1.1-260L; however, we failed to rescue clonal rgBQ.1.1-260L because it replicated remarkably slowly *in vitro,* and the rescued viruses acquired additional mutations (e.g., NSP3-Y94H, NSP3-N1220K, NSP5-P96L, NSP9-T21I, and NSP10-N105K) that may have influenced its replicative ability. These findings indicate that NSP6-L260F is essential for BQ.1.1 replication. Although we could not isolate clonal rgBQ.1.1-260L, one of the rescued rgBQ.1.1-260L possessed only one additional substitution, NSP9-T21I. We, therefore, generated rgBQ.1.1 with NSP9-T21I (rgBQ.1.1-260F-NSP9-T21I) and rgBQ.1.1-NSP9-T21I lacking NSP6-L260F (rgBQ.1.1-260L-NSP9-T21I) and used these viruses for subsequent analyses (Fig. 1b). To compare the growth kinetics of these viruses *in vitro*, VeroE6/TMPRSS2 cells were infected with each virus at a multiplicity of infection (MOI) of 0.0001. The virus titers were determined at 6, 12, 24, and 48 h post-infection (hpi). In rgXBB.1.5-backbone viruses, rgXBB.1.5-260F showed higher virus titers at 24 and 48 hpi than rgXBB.1.5-260L (Fig. 1c). In rgBQ.1.1-NSP9-T21I-backbone viruses, rgBQ.1.1-260L-NSP9-T21I replicated less efficiently than rgBQ.1.1-260F-NSP9-T21I (Fig. 1c). There were no significant differences in virus replication between rgBQ.1.1-260L and rgBQ.1.1-260L-NSP9-T21I, suggesting that the NSP9-T21I substitution did not affect virus replication under these conditions. In addition, VeroE6/TMPRSS2 cells were infected with rgXBB.1.5-260L (WT), rgXBB.1.5-260F, rgBQ.1.1-260F-NSP9-T21I, or rgBQ.1.1-260L-NSP9-T21I at an MOI of 0.001 in triplicate, and viruses collected at 24 and 48 hpi were deep sequenced. Viruses with revertant mutations or additional mutations were not detected (data not shown).

Next, we compared the replicative fitness of viruses with NSP6-260L and viruses with NSP6-260F by performing a competition assay. rgXBB.1.5-260L (WT) and rgXBB.1.5-260F or rgBQ.1.1-260F-NSP9-T21I and rgBQ.1.1-260L-NSP9-T21I were mixed at an equal ratio on the basis of their infectious titer, and the virus mixture was infected into VeroE6/TMPRSS2 cells at an MOI of 0.001. At 24 and 48 hpi, the supernatant containing viruses was collected and assessed by deep sequencing analysis to determine the proportion of each virus. The variant possessing the NSP6-260F substitution became predominant by 48 hpi in either the XBB.1.5 backbone or the BQ.1.1-NSP9-T21I backbone (Fig. 1d). When the virus was passaged every 24 h after co-infection, the proportion of the variant with NSP6-260L became less than 1% or 3% in the XBB.1.5 backbone or the BQ.1.1-NSP9-T21I backbone, respectively (Fig. 1e).

### NSP6-L260F enhances virus pathogenicity in hamsters

**Fig 2:**
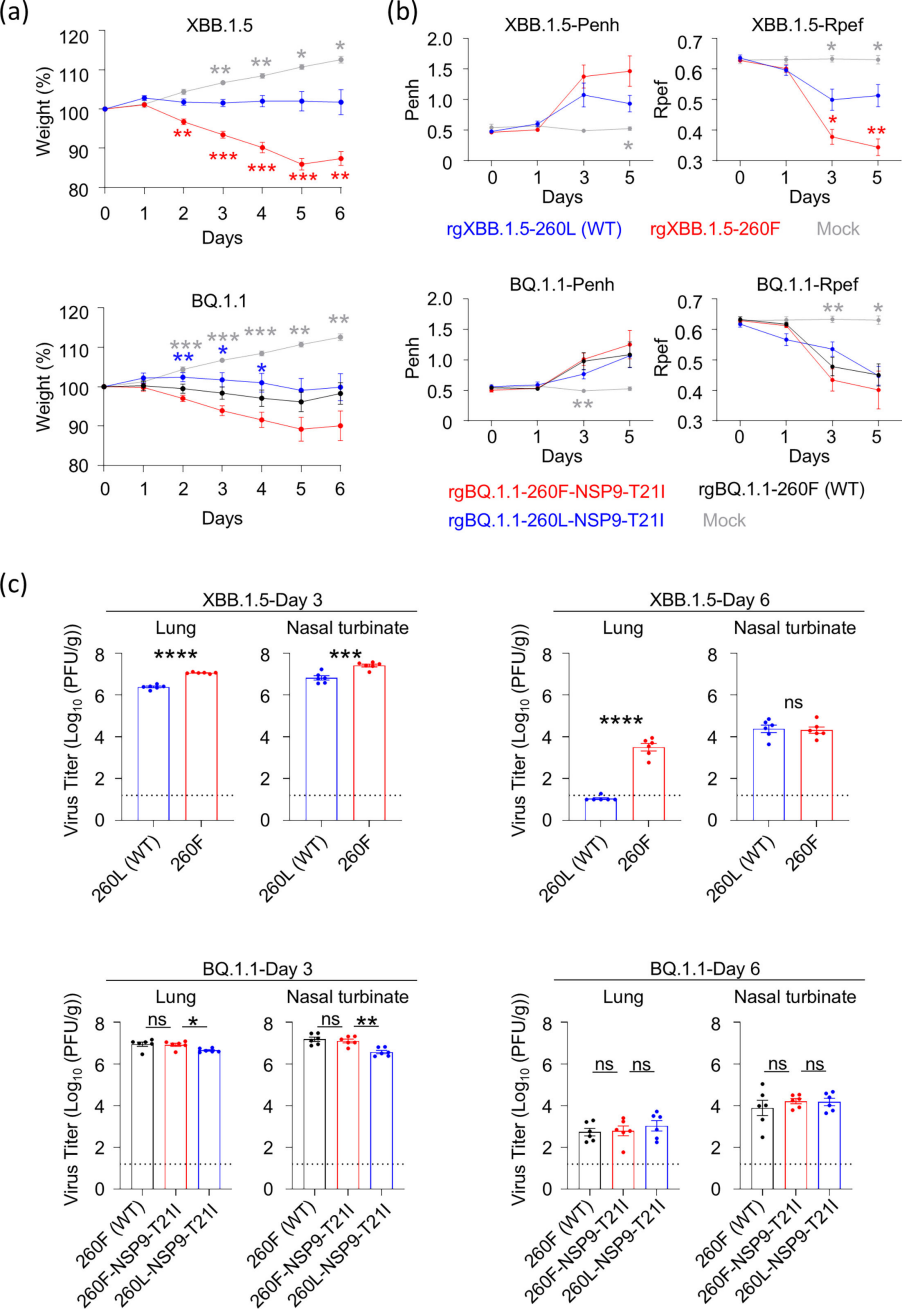
The effect of NSP6-L260F on viral *in vivo* characteristics. Syrian hamsters were intranasally inoculated with 10^5^ PFU (in 100 µL) of the indicated virus. (**a**) Body weights of virus-infected and mock-infected hamsters (*n* = 5) were monitored daily for 6 days. Data are presented as the mean percentages of the starting weight (± s.e.m.). (**b**) Pulmonary function analyses in infected hamsters. Penh and Rpef were measured by using whole-body plethysmography (*n* = 5, mean ± s.e.m.). (**c**) Virus titers in infected Syrian hamsters. Hamsters (*n* = 5) were euthanized at 3 and 6 days post-infection for virus titration. Virus titers in the nasal turbinate and lungs were determined by using plaque assays. Vertical bars show the mean ± s.e.m. Points indicate data from individual hamsters. The lower limit of detection is indicated by the horizontal dashed line. Data were analyzed by using a two-way ANOVA with Dunnett’s multiple comparisons test (a and b), *t*-test (c, XBB.1.5-backbone), or a one-way ANOVA with Dunnett’s multiple comparisons test (c, BQ.1.1-backbone). Statistical significance between rgXBB.1.5-260L (WT) and rgXBB.1.5-260F is shown by red asterisks, that between rgBQ.1.1-260F-NSP9-T21I and rgBQ.1.1-260L-NSP9-T21I by blue asterisks, and that between rgXBB.1.5-260L (WT) or rgBQ.1.1-260F-NSP9-T21I and mock by gray asterisks. ∗*P* < 0.05, ∗∗*P* < 0.01, ∗∗∗*P* < 0.001, ∗∗∗∗*P* < 0.0001, and ns; not significant.

We then assessed the effect of NSP6-L260F on pathogenicity in Syrian hamsters, a well-established animal model for the study of SARS-CoV-2 (13–15). Hamsters were intranasally inoculated with 10^5^ plaque-forming units (PFUs) of each virus. Mock-infected animals gained weight over the 6-day experiment (Fig. 2a). Animals infected with rgXBB.1.5-260L (WT) did not gain weight, whereas intranasal infection with rgXBB.1.5-260F resulted in significant body weight loss by 6 days post-infection. rgBQ.1.1-260F-NSP9-T21I also caused body weight loss; however, intranasal infection with rgBQ.1.1-260L-NSP9-T21I resulted in significantly less body weight loss compared with rgBQ.1.1-260F-NSP9-T21I (Fig. 2a). In addition, hamsters infected intranasally with rgBQ.1.1-260F (WT) showed less body weight loss than those infected with rgBQ.1.1-260F-NSP9-T21I. However, the difference in body weight change between rgBQ.1.1-260F (WT)- and rgBQ.1.1-260F-NSP9-T21I-infected animals was not statistically significant.

We next evaluated the pulmonary functions Penh (enhanced pause) and Rpef (peak expiratory flow rate) in the infected hamsters by using the whole-body plethysmography system (Fig. 2b). No statistically significant differences in the Penh of hamsters infected with rgXBB.1.5-backbone viruses or in the Penh or Rpef of hamsters infected with rgBQ.1.1-backbone viruses were observed between all groups. However, rgXBB.1.5-260F caused a significant reduction in Rpef and a slight increase in Penh compared with rgXBB.1.5-260L (WT) on days 3 and 5 post-infection.

On days 3 and 6 post-infection, virus titers in the nasal turbinate and lungs of hamsters infected with each virus were measured by using plaque assays (Fig. 2c). On day 3 post-infection, NSP6-260F significantly increased virus titers in the lungs and nasal turbinates. On day 6 post-infection, no differences were observed in the hamsters infected with rgBQ.1.1-backbone viruses, whereas rgXBB.1.5-260F titers were significantly higher in the lungs than rgXBB.1.5-260L (WT) titers. These results suggest that NSP6-260F enhances virus replication and pathogenicity in hamsters. In addition, rgBQ.1.1-260F (WT)-infected hamsters showed a similar weight change to hamsters infected with rgBQ.1.1-260L-NSP9-T21I, but not rgBQ.1.1260F-NSP9-T21I-infected hamsters, even though the replicative ability of rgBQ.1.1-260F (WT) was similar to that of rgBQ.1-260F-NSP9-T21I both *in vitro* and *in vivo* (Fig. 1c and 2c). This result suggests that NSP9-T21I might increase viral pathogenicity slightly, but not significantly.

### NSP6-L260F promotes viral RNA replication in cells

**Fig 3:**
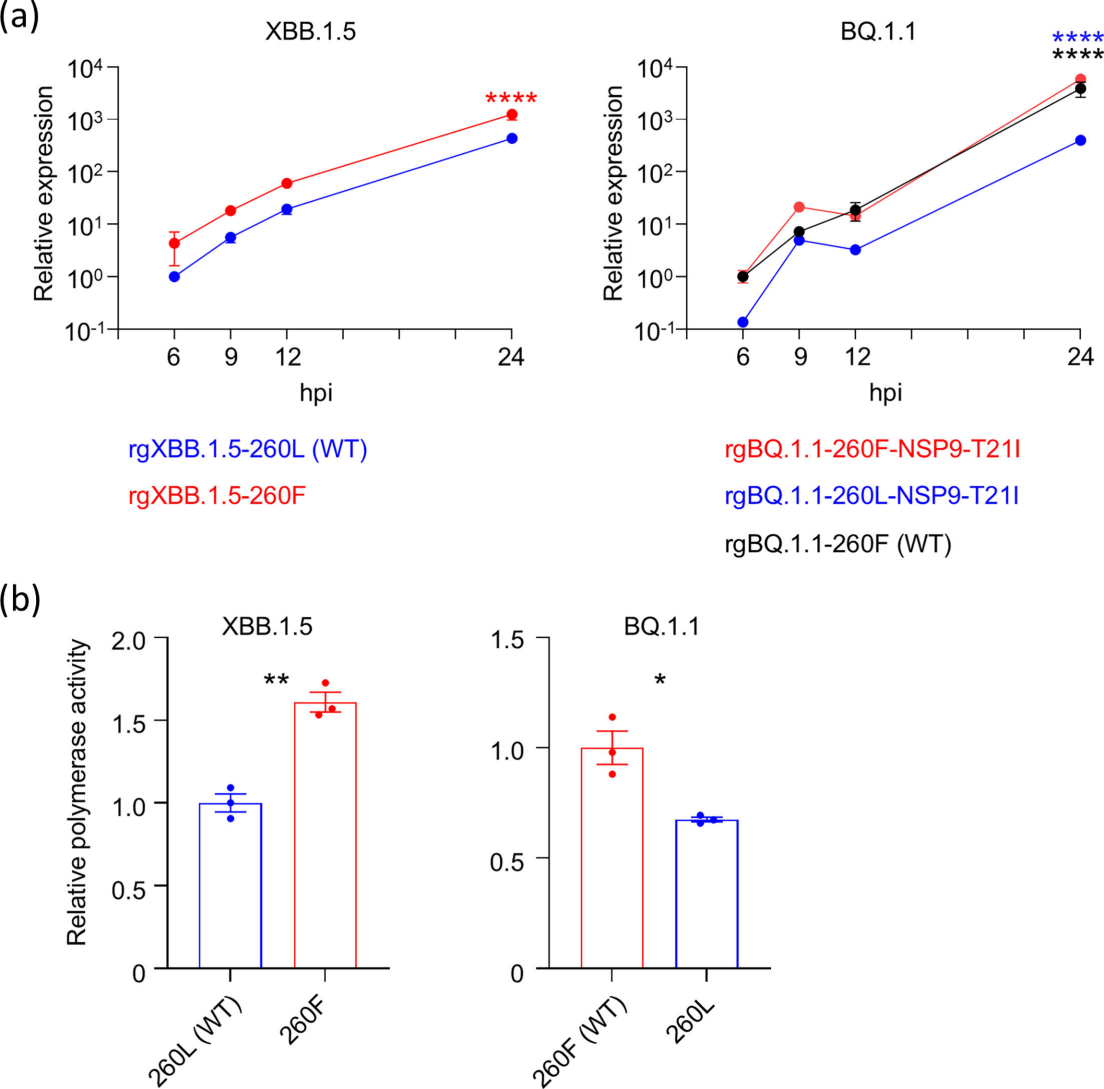
The role of NSP6-L260F in virus transcription/replication. (**a**) VeroE6/TMPRSS2 cells were infected with each virus at an MOI of 1. At the indicated time points, total RNA was extracted from the infected cells, and the relative expression of sgRNA for the E gene was measured by RT-qPCR (*n* = 3, mean ± s.e.m.). Data were analyzed by using a two-way ANOVA with Dunnett’s multiple comparisons test. Statistical significance between rgXBB.1.5-260L (WT) and rgXBB.1.5-260F is shown by red asterisks, that between rgBQ.1.1-260F-NSP9-T21I and rgBQ.1.1-260L-NSP9-T21I by blue asterisks, and that between rgBQ.1.1-260F-NSP9-T21I and rgBQ.1.1-260F (WT) by black asterisks. (**b**) Polymerase activity in HEK293T cells transfected with the replicative cDNA containing the nanoluciferase gene was determined at 72 h post-transfection (*n* = 3, mean ± s.e.m.). The results are representative of three independent experiments. Data were analyzed by using a *t*-test. ∗*P* < 0.05, ∗∗*P* < 0.01, ∗∗∗*P* < 0.001, and ∗∗∗∗*P* < 0.0001.

In SARS-CoV-2-infected cells, RNA transcription and replication take place in DMVs (5, 16, 17). DMVs are tethered to the ER by membrane connectors. NSP6 zippers ER membranes and forms the connectors (4, 5), suggesting that NSP6 might affect viral RNA replication. We, therefore, quantified the expression of the sub-genomic RNA (sgRNA) of the Envelope gene, which can be used as an indicator of active transcription/replication. VeroE6/TMPRSS2 cells were infected with each virus at an MOI of 1. Total RNA was extracted from infected cells at the indicated time points, and the amount of E-sgRNA was measured by quantitative reverse transcription-PCR (RT-qPCR). The expression of E-sgRNA in cells infected with rgXBB.1.5-260F was higher than that in cells infected with rgXBB.1.5-260L (Fig. 3a). Similar results were observed with BQ.1.1-backbone viruses. In addition, we assessed polymerase activity by using replicon DNAs, which lack the structural S, M, and E genes and contain the nanoluciferase gene between the replicase and N genes (18). Replicon DNAs of XBB.1.5- and BQ.1.1-backbone constructs with NSP6-260F or NSP6-260L were transfected into HEK293T cells, and luciferase activity, as a readout of polymerase activity, was measured 3 days post-transfection. The results showed that NSP6-260F significantly enhances polymerase activity in XBB.1.5- and the BQ.1.1-backbone experiments (Fig. 3b).

### The amino acid substitution at position 260 does not affect the inhibitory activity of NSP6 on interferon production and responses

**Fig 4:**
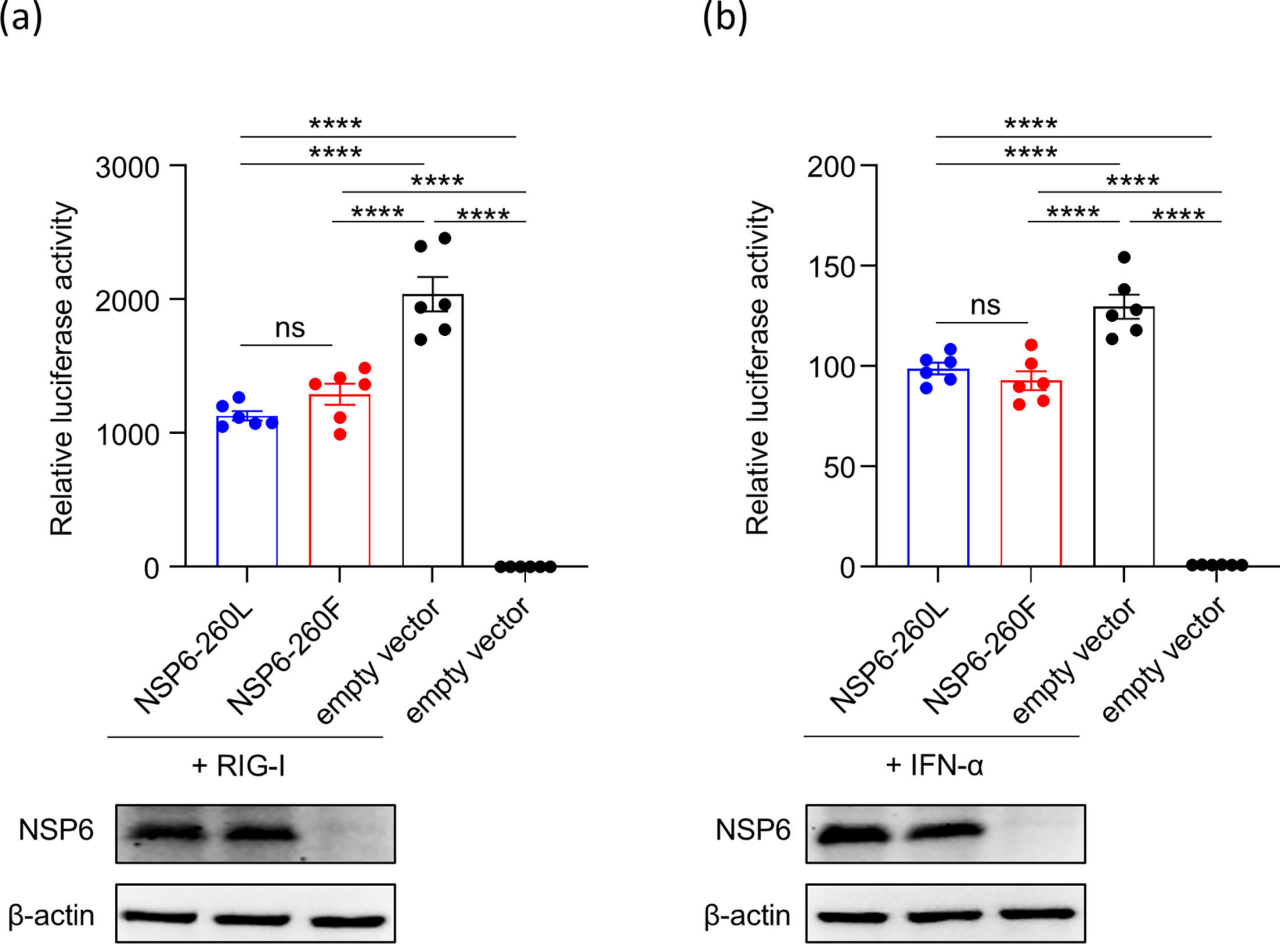
The role of NSP6-L260F in IFN responses. (**a**) NSP6 inhibition of RIG-I-dependent IFN-β production. HEK293T cells were transfected with the indicated viral protein expression plasmids, p125-luc, and pGL4.74 [hRluc/TK] vector, with or without a plasmid encoding the constitutively active mutant N-Myc-RIG-IN. IFN-β promoter activity was calculated by normalizing the firefly luciferase activity to the *Renilla* luciferase activity. The IFN-β promoter activity without N-Myc-RIG-IN was set to 1. The data are shown as mean relative IFN-β promoter activities (*n* = 3, mean ± s.e.m.). (**b**) NSP6 inhibition of type-I IFN signaling. HEK293T cells were transfected with the indicated viral protein expression plasmids, pISRE-luc, and pGL4.74 [hRluc/TK] vector. Eight hours after treatment with IFN-α, firefly and *Renilla* luciferase activities were measured by using a dual-luciferase assay. ISRE-driven firefly luciferase activity was calculated by normalization to the *Renilla* luciferase activity. The firefly luciferase activity without IFN-α was set to 1. The data are shown as mean relative firefly luciferase activities (*n* = 3, mean ± s.e.m.). NSP6 expression was confirmed by western blotting. (a and b) The results are representative of three independent experiments. Data were analyzed by using a one-way ANOVA with Dunnett’s multiple comparisons test. ∗∗∗∗*P* < 0.0001, ns; not significant.

In addition to forming connectors for DMVs, NSP6 antagonizes the type I interferon (IFN) response by inhibiting IFN-β induction and IFN signaling (5, 19). Therefore, we evaluated the effect of NSP6-L260F on IFN production and response. We cloned the NSP6 of BQ.1.1 or its mutant with an N-terminal FLAG tag into a mammalian expression plasmid. The N-terminal Flag tag does not alter the localization of NSP6, and the N-terminally tagged NSP6 retains its ability to form connectors between the ER and DMVs (4). To assess their inhibitory effect on RIG-I-dependent IFN-β production, we co-transfected HEK293T cells with four plasmids: (i) a plasmid expressing NSP6 or an empty pCAGGS vector; (ii) pCA-N-Myc-RIG-IN, which encodes a constitutively active mutant of human RIG-I; (iii) p125-luc, which contains the IFN-promoter to drive the expression of firefly luciferase; and (iv) a control plasmid pGL4.74[hRluc/TK] (phRluc-TK) to normalize transfection efficiency (19, 20). At 24 h post-transfection, luciferase activities in the transfected cells were measured to quantify the IFN-β promoter activity. Compared to the empty vector control, NSP6 suppressed IFN-β promoter activity as previously reported (19). However, no differences were observed between NSP6-260F and NSP6-260L (Fig. 4a). We also evaluated the ability of NSP6-260F and NSP6-260L to antagonize IFN signaling. HEK293T cells were co-transfected with three plasmids: (i) a plasmid expressing NSP6 or the empty pCAGGS vector; (ii) pISRE-luc, which contains a luciferase reporter driven by an interferon-stimulated response element (ISRE) promoter; and (iii) a control plasmid pGL4.74[hRluc/TK] (phRluc-TK) to normalize transfection efficiency (19). At 24 h post-transfection, the transfected cells were treated with IFN-α for 8 h and assayed for luciferase signals to quantify the activation of ISRE. Compared to the empty vector control, NSP6 suppressed IFN signaling as previously reported (19). However, no differences were observed between NSP6-260F and NSP6-260L (Fig. 4b). These results suggest that NSP6-L260F does not affect the ability of NSP6 to antagonize IFN production and responses.

### NSP6-L260F compensates for disadvantageous mutations

**Fig 5:**
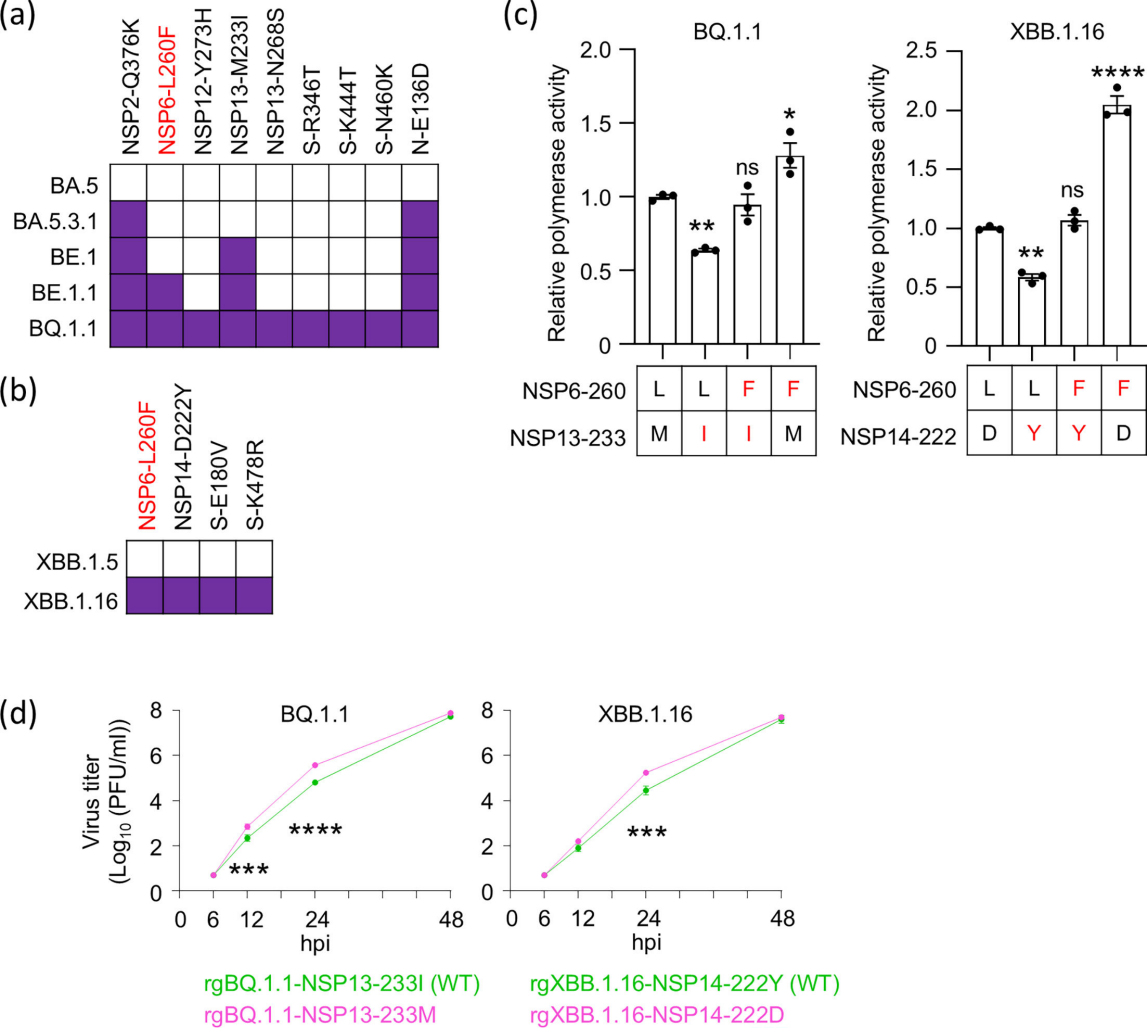
Identification of disadvantageous mutations that are compensated for by NSP6-L260F. (a and b) Amino acid residues that differ between the indicated lineages. (**c**) The effect of amino acid combinations on polymerase activity. Polymerase activity in HEK293T cells transfected with the replicon DNA containing the nanoluciferase gene was determined at 72 h post-transfection (*n* = 3, mean ± s.e.m.). Amino acids that are the same as the prototype are shown in black, and mutations that BQ.1.1 or XBB.1.16 carry are shown in red. Data were analyzed by using a one-way ANOVA with Dunnett’s multiple comparisons test. Statistical significance was calculated against the values when the amino acids in both positions were the same as in the prototype. ∗*P* < 0.05, ∗∗*P* < 0.01, ∗∗∗*P* < 0.001, ∗∗∗∗*P* < 0.0001, and ns, not significant. The results are representative of three independent experiments. (**d**) VeroE6/TMPRSS2 cells were infected with the indicated virus at an MOI of 0.0001. Virus titers at the indicated time points were determined by using plaque assays (*n* = 3, mean ± s.e.m.). Data were analyzed by using a two-way ANOVA with Dunnett’s multiple comparisons test. ∗∗∗*P* < 0.001 and ∗∗∗∗*P* < 0.0001.

Although NSP6-L260F promotes viral replication, it is only found in a limited number of lineages, such as BQ.1.1 and XBB.1.16. As mentioned above, clonal rgBQ.1.1-260L could not be rescued. Furthermore, the generation of the XBB.1.16 virus possessing NSP6-260L also failed. These results indicate that NSP6-260L in the BQ.1.1- or XBB.1.16-backbone downregulates polymerase activity. However, NSP6-260L is found in most clinical isolates, suggesting that amino acid substitutions specific to BQ.1.1 and/or XBB.1.16 reduce virus polymerase activity. To identify candidate substitutions that reduce polymerase activity, we compared the amino acid sequences of BQ.1.1 and XBB.1.16 with their closely related lineages. BQ.1.1 evolved from BA.5 through BA.5.3.1, BE.1, and BE.1.1 (Fig. 5a). The NSP6-L260F substitution appeared in the BE.1.1 lineage and was maintained in its progeny BQ.1.1 lineage, suggesting that the disadvantageous substitution was likely introduced upon evolution from BA.5 to BE.1.1. During this process, the three substitutions NSP2-Q376K, NSP13-M233I, and N-E136D were acquired (Fig. 5a). When we looked at viral proteins (NSP3–NSP16) that are directly linked to NSP6 functions, such as polyprotein cleavage, DMV formation, RNA transcription, and RNA replication, NSP13-M233I (ORF1b-M1156I) was a candidate substitution responsible for reducing polymerase activity in the BQ.1.1 lineage. Next, we compared the amino acid sequences of XBB.1.5 and XBB.1.16 since XBB.1.16 evolved from XBB.1.5 (Fig. 5b). We found three additional amino acid differences (NSP14-D222Y, S-E180V, and S-K478R) (Fig. 5b). NSP14-D222Y (ORF1b-D1746Y) is a candidate substitution responsible for reducing polymerase activity in the XBB.1.16 lineage. We, therefore, examined whether the NSP13-M233I and NSP14-D222Y substitutions are involved in reduced polymerase activity. Replicon DNAs with different combinations of amino acids at NSP6-260 and NSP13-233 or NSP14-222 in the BQ.1.1- or XBB.1.16-backbone were constructed, and their polymerase activity was compared *in vitro*. Polymerase activity with the prototype amino acids at each position (i.e., NSP6-260L and NSP13-233M for BQ.1.1 and NSP6-260L and NSP14-222D for XBB.1.16) was set to 1. In the BQ.1.1-backbone, the NSP13-M233I substitution significantly reduced the polymerase activity, and the NSP6-L260F substitution restored it (Fig. 5c). In the XBB.1.6-backbone, the NSP14-D222Y substitution significantly reduced the polymerase activity, and the NSP6-L260F restored it (Fig. 5c). These results show that the NSP13-M233I and NSP14-D222Y substitutions reduce viral polymerase activity. To confirm this adverse effect, we generated viruses with or without these substitutions, that is, rgBQ.1.1-NSP13-233I (WT), rgBQ.1.1-NSP13-233M, rgXBB.1.16-NSP14-222Y (WT), and rgXBB.1.16-NSP14-222Y, and compared their replicative ability in VeroE6/TMPRSS2 cells. Similar to the replicon assays, virus replication was significantly (albeit not at all time points tested) increased by removing the disadvantageous substitutions NSP13-M233I and NSP14-D222Y (Fig. 5d). These results suggest that NSP13-M233I in BQ.1.1 and NSP14-D222Y in XBB.1.16 decrease virus replication and that NSP6-L260F compensates for this.

## DISCUSSION

Here, we showed that NSP6-L260F promotes virus replication *in vitro* and *in vivo* and virus polymerase activity *in vitro*. This substitution is found in human isolates, including those of the BQ.1.1 and XBB.1.16 lineages and has been reported in several papers (21–27). Although a previous study suggested that NSP6-L260F is under positive selection in human isolates (27), its effects on viral characteristics have not been fully explored. Since NSP6 is important for the biogenesis of the SARS-CoV-2 replication organelle (4), we examined virus polymerase activity and replication efficiency *in vitro* and found that NSP6-L260F-induced promotion of virus replication is achieved by increasing RNA polymerase activity. The structure of NSP6 has not been determined, and the predicted structure is controversial; it may have six transmembrane domains and an amphipathic helix that does not cross the membrane but associates with it (4, 5, 28), or seven transmembrane domains (29, 30), or eight transmembrane domains (31, 32). However, NSP6-260 is predicted to be located in the C-terminal region of NSP6 in all prediction models. The C-terminal region is thought to be important for interactions with lipid droplets required for SARS-CoV-2 replication (4). NSP6 induces lipid flux into DMVs (4, 7). Therefore, NSP6-L260F may enhance the interaction with lipid droplets or lipid flux activity to promote RNA replication in DMVs. Recently, another group found that NSP6-L260F enhances viral RNA replication and pathogenicity, consistent with our findings (33). Furthermore, they revealed that NSP6-L260F reduces lipid content in host cells, supporting our hypothesis. Although we did not look at changes in DMV formation, the main known function of NSP6 is ER zippering and transport of proteins and lipids into DMVs (4). DMVs are formed by NSP3 and NSP4, and NSP6 is not required for DMV formation. It has also been reported that NSP6-L260F does not alter the subcellular localization of NSP6 (33). Furthermore, a similar mutation in the C-terminal region of MERS NSP6 (L232F) enhances RNA replication without altering DMV morphology (34). Taken together, these findings support our hypothesis that NSP6-L260F enhances RNA replication by affecting lipid consumption.

We also identified substitutions in the BQ.1.1 and XBB.1.16 lineages that reduce virus replication in VeroE6/TMPRSS2 cells: NSP13-M233I in BQ.1.1 and NSP14-D222Y in XBB.1.16. These substitutions also reduce polymerase activity. The introduction of the NSP6-L260F substitution restores the polymerase activity, suggesting that it compensates for the negative effects of these disadvantageous substitutions. When we looked at the evolution of BA.5 to BQ.1.1, we saw that NSP13-M233I first appeared in the BE.1 lineage, and right after that, NSP6-L260F appeared in the BE.1.1 lineage (Fig. 5a). When we examined the amino acid changes from XBB.1.5 to XBB.1.16 using COV2Var, a functional annotation database of SARS-CoV-2 (27), we found that the number of sequences carrying NSP14-D222Y increased at the beginning of 2023 and was higher than that carrying NSP6-L260F at that time. This indicates that NSP14-D222Y appeared earlier than NSP6-L260F in the XBB.1 lineage and supports our hypothesis that NSP6-L260F may be acquired to compensate for the adverse effects of NSP13-M233I or NSP14-D222Y. It is intriguing that NSP6-L260F, which promotes viral growth, has not become a consensus substitution. Possibly, NSP6-L260F is selected only in the presence of substitutions that are unfavorable for viral polymerase activity, such as NSP13-M233I and NSP14-D222Y, although why such substitutions were present in these viruses remains unclear.

The increased replicative ability caused by NSP6-L260F enhanced virus pathogenicity in hamsters. It caused more body weight loss and a greater reduction in respiratory functions. Previously, NSP6-L260F was identified as an amino acid substitution that correlates with higher hospitalization rates (27). Another report found that infection with XBB.1.16, which naturally possesses NSP6-L260F, leads to a significantly higher hospitalization rate than other Omicron variants (12). Therefore, NSP6-L260F may increase viral pathogenicity in humans, although viral pathogenicity in humans is difficult to evaluate because it is influenced by other factors, including immunity and social situations. In addition, our results suggest that NSP9-T21I might increase viral pathogenicity slightly. The role of NSP9-T21I in virus pathogenicity requires further characterization, as NSP9 has the potential to affect innate immunity (35, 36). Furthermore, the effect of F260L on BQ.1.1-NSP9-T21I is more moderate than that of L260F on XBB.1.5 in the hamster model; this difference might be caused by the additional acquisition of NSP9-T21I.

NSP13-M233I and NSP14-D222Y downregulated virus polymerase activity *in vitro*. NSP13 belongs to the helicase superfamily 1B and catalyzes the unwinding of double-stranded DNA or RNA (37). It interacts with NSP12 (viral RNA-dependent RNA polymerase) and functions as part of the replication-transcription complex with NSP7, NSP8, and NSP12 (38). NSP13 has five domains (zinc binding domain, stalk, 1B, 1A, and 2A) (39), and NSP13-233 is located in the 1B domain; however, its role is unclear. NSP14 is a multifunctional protein that contains an exoribonuclease domain involved in proofreading and a guanine N7 methyl transferase involved in mRNA capping (40–43). NSP14-222 is located in the exoribonuclease domain, but its exact role has not been determined. Therefore, the reason why these disadvantageous substitutions accumulated in the viral genome remains unknown. NSP13-M233I and NSP14-D222Y increase protein stability (27, 44), and NSP14-D222Y was positively selected in the XBB.1 lineage (27), suggesting that they may have some beneficial roles in virus survival in humans, even though they decreased virus replication in VeroE6/TMPRSS2 cells.

In conclusion, we found that NSP6-L260F promotes virus replication *in vitro* and *in vivo* by increasing polymerase activity and enhances virus pathogenicity in hamsters. This finding is consistent with a recent report by another group (33). We also identified substitutions that reduce polymerase activity in BQ.1.1 and XBB.1.16 that are compensated for by NSP6-L260F. The acquisition of NSP6-L260F or the loss of the suppressive mutations in BQ.1.1 and XBB.1.16, which could increase virus pathogenicity, should be monitored carefully. Given that the NSP6-L260F substitution promotes virus replication in cultured cells, this substitution could be used to improve the production of inactivated or live attenuated vaccine virus.

## MATERIALS AND METHODS

### Cells

VeroE6/TMPRSS2 (JCRB 1819) cells (45, 46) were propagated in the presence of 1 mg/mL geneticin (G418; Invivogen) and 5 µg/mL Plasmocin Prophylactic (InvivoGen) in Dulbecco’s modified Eagle’s medium (DMEM) containing 10% fetal bovine serum (FBS). HEK293T cells were cultured in DMEM supplemented with 10% FBS. All cells were maintained at 37°C with 5% CO_2_. The cells were regularly tested and confirmed to be negative for mycoplasma contamination by using PCR.

### Bacterial artificial chromosome construction

Six overlapping DNA fragments spanning the whole SARS-CoV-2 genome were amplified by PCR using PrimeSTAR GXL DNA Polymerase (TaKaRa). cDNAs synthesized from the following viruses were used as templates for the PCR: hCoV-19/USA/MD-HP40900-PIDYSWHNUB/2022 (Omicron, XBB.1.5) (47–49), hCoV-19/Japan/TY41-796/2022 (Omicron, BQ.1.1) (9, 10, 50), and hCoV-19/USA/un-UOT-IRI97754/2023 (Omicron, XBB.1.16). Note that XBB.1.16 has an additional S-R682G mutation compared to the sequence registered in the GISAID database, which seems to have been introduced during virus propagation. Mutations in NSP6, NSP13, or NSP14 were introduced into fragments by using primer-guided mutagenesis. The six fragments and the linearized pBeloBAC11 vector were assembled by using NEBuilder HiFi DNA Assembly Master Mix (NEB), resulting in infectious cDNA clones under the control of a cytomegalovirus promoter. ET SSB (NEB) was added to the reaction mix to improve the efficiency and accuracy of the assembly reaction (51). The constructed bacterial artificial chromosomes (BACs) were introduced into ElectroMAX DH10B Cells (NEB) by electroporation. *Escherichia coli* was amplified at 37°C, and BACs were extracted by using NucleoBond Xtra Maxi (TaKaRa). The sequences of all constructs were confirmed by Sanger sequencing.

### Recombinant SARS-CoV-2 generation

To recover recombinant SARS-CoV-2, BACs encoding the full-length SARS-CoV-2 genome were transfected into HEK293T cells using TransIT-293 (TaKaRa) according to the manufacturer’s protocol. At 3 days post-transfection, the supernatant containing viruses was collected and inoculated onto VeroE6/TMPRSS2 cells at 37°C to prepare the virus stock. The virus titers of the stock viruses were determined by performing plaque assays with VeroE6/TMPRSS2 cells. The stock viruses were deep sequenced as described below to confirm that there were no unintended amino acid substitutions with a frequency greater than 10% in any of the virus stocks.

### Deep sequencing analysis

Viral RNA was extracted by using a QIAamp Viral RNA Mini Kit (Qiagen) and RNase-free DNase Set (Qiagen). The whole genome of SARS-CoV-2 was amplified by using a modified ARTIC network protocol in which some primers were replaced or added (52). Briefly, viral cDNA was synthesized from the extracted RNA by using a LunarScript RT SuperMix Kit (New England BioLabs). The DNA was amplified by performing a multiplexed PCR in two pools using the ARTIC-N6 primers and the Q5 High-Fidelity DNA polymerase or Q5 Hot Start DNA polymerase (New England BioLabs). DNA libraries for Illumina NGS were prepared from pooled amplicons by using a QIAseq FX DNA Library Kit (Qiagen) and were then analyzed by using the iSeq 100 System (Illumina). To determine the virus sequences, the reads were assembled by CLC Genomics Workbench (version 23, Qiagen) with the Wuhan/Hu-1/2019 sequence (GenBank accession no. MN908947) as a reference.

### Growth kinetics in cultured cells

VeroE6/TMPRSS2 cells grown on 24-well plates in triplicate were infected with the indicated virus at a multiplicity of infection of 0.0001. The inoculum was removed after 60 min of incubation at 37°C, and the cells were then further incubated at 37°C. Cell culture supernatants were collected at the indicated time points. Virus titers were determined by performing a plaque assay with VeroE6/TMPRSS2 cells.

### Competition assay

rgXBB.1.5-260L (WT) and rgXBB.1.5-260F or rgBQ.1.1-260F-NSP9-T21I and rgBQ.1.1-260L-NSP9-T21I were mixed at an equal ratio on the basis of their infectious titer, and the virus mixture was infected into VeroE6/TMPRSS2 cells grown on 24-well plates in triplicate at an MOI of 0.001. At 24 and 48 hpi, the supernatant containing the viruses was collected. Virus passage was performed as described previously (53). At 24 hpi, 100 µL of infected cell culture supernatant was transferred to fresh VeroE6/TMPRSS2 cells for four subsequent passages. Viral RNA was isolated from each culture supernatant by using a QIAamp Viral RNA Mini Kit (Qiagen) and RNase-free DNase Set (Qiagen) and was assessed by deep sequencing analysis to determine the proportion of each virus.

### Animal experiments

Virus inoculations were performed under anesthesia, and all efforts were made to minimize animal suffering. *In vivo* studies were not blinded, and animals were randomly assigned to infection groups. No sample-size calculations were performed to power each study. Instead, sample sizes were determined based on prior *in vivo* virus challenge experiments.

### Experimental infection of Syrian hamsters

Five- to six-week-old male wild-type Syrian hamsters (Japan SLC Inc., Shizuoka, Japan) were used in this study. Baseline body weights were measured before infection. Under ketamine-xylazine anesthesia, six hamsters per group were intranasally inoculated with 10^5^ PFU (in 100 µL) of the indicated virus. Body weight was monitored daily for 6 days. For virological examinations, 12 hamsters per group were intranasally infected with 10^5^ PFU (in 100 µL) of the indicated virus; 3 and 6 days post-infection, six animals per group were euthanized, and nasal turbinates and lungs were collected and homogenized. The virus titers in the nasal turbinate and lung samples were determined by using plaque assays in VeroE6/TMPRSS2 cells.

### Measuring lung function

Respiratory parameters were measured by using a whole-body plethysmography system (PrimeBioscience) according to the manufacturer’s instructions. In brief, hamsters were placed in the unrestrained plethysmography chambers and allowed to acclimatize for 1 min before data were acquired over a 3-min period by using FinePointe software.

### Subgenomic viral RNA qRT-PCR

VeroE6/TMPRSS2 cells grown on 24-well plates in triplicate were infected with the indicated virus at an MOI of 1 and incubated at 37°C. Total RNA was extracted from infected cells at the indicated time points by using an RNeasy Mini kit (Qiagen) and RNase-free DNase Set (Qiagen). The extracted RNA was reverse-transcribed into cDNA and amplified by using the QuantiTect Probe RT-PCR kit (Qiagen). Quantitative reverse-transcription PCR was performed and analyzed on a Light Cycler 96 System (Roche). The cycle conditions of RT-qPCR were 50°C for 30 min, 95°C for 15 min, followed by 45 cycles of 95°C for 15 s, and 60°C for 60 s. The expression of E-sgRNA was analyzed by using the 2^–ΔΔCT^ method (54) and normalized to the expression of GAPDH mRNA (55). The primer and probe sequences were as follows: E-sgRNA-F: CGATCTCTTGTAGATCTGTTCTC, E-sgRNA-R: ATATTGCAGCAGTACGCACACA, E-sgRNA-probe: FAM-ACACTAGCCATCCTTACTGCGCTTCG-BHQ1 (56), GAPDH-F: GAAGGTGAAGGTCGGAGTC, GAPDH-R: GAAGATGGTGATGGGGCTTC, and GAPDH-probe: FAM-CAAGCTTCCCGTTCTCAGCC-BHQ1 (57).

### Replicon assay

The replicative cDNA of SARS-CoV-2, which lacks the structural S, M, and E genes and contains the nanoluciferase gene between the replicase and N genes with a transcription regulatory sequence of the M gene, was reported previously (18). The replicative cDNAs of hCoV-19/USA/MD-HP40900-PIDYSWHNUB/2022 (Omicron, XBB.1.5) (47–49), hCoV-19/Japan/TY41-796/2022 (Omicron, BQ.1.1) (9, 10, 50), and hCoV-19/USA/un-UOT-IRI97754/2023 (Omicron, XBB.1.16) were assembled into pBeloBAC11 as described above. For the replicon assay, HEK293T cells were seeded onto 24-well plates at 1.5 × 10^5^ cells/well and cultured at 37°C overnight. The BACs encoding the replicative cDNA and pGL3-control vector were transfected into HEK293T cells using TransIT-LT1 (TaKaRa) according to the manufacturer’s instructions. pGL3 Control Vector, a gift from Debrya Groskreutz (Addgene plasmid #212937; http://n2t.net/addgene:212937; RRID: Addgene_212937), expressed firefly luciferase and served as an internal control. After incubation for 72 h at 37°C, the transfected cells were lysed with Passive Lysis buffer (Promega). The nanoluciferase activity and the firefly luciferase activity were measured by using the Nano-Glo luciferase assay system (Promega) and Luciferase Assay System (Promega), respectively. Polymerase activity was calculated by normalizing the nanoluciferase activity to the firefly luciferase activity.

### Plasmid construction

The open reading frames of BQ.1.1-NSP6 or BQ.1.1-NSP6-F260L with a Flag-tag at the N-terminal, start, and stop codons were subcloned into pCAGGS/MCS for protein expression.

### IFN-β promoter reporter assay

The IFN-β promoter reporter assay was performed as previously reported (20), with some modifications. HEK293T cells were seeded onto 24-well plates and cultured at 37°C overnight. The cells were then transfected with the indicated viral protein expression plasmids or pCAGGS empty vector (500 ng), p125-luc (100 ng; kindly provided by T. Fujita, Kyoto University) (58) containing the IFN-β promoter, which drives the expression of firefly luciferase, and pGL4.74 [hRluc/TK] vector (50 ng; Promega), which served as a transfection control, with or without a plasmid encoding the constitutively active mutant N-Myc-RIG-IN (20) (50 ng), by using Trans-IT 293. At 24 h post-transfection, firefly and *Renilla* luciferase activities were measured by using the Dual-Glo luciferase assay system (Promega). IFN-β promoter activity was calculated by normalizing the firefly luciferase activity to the *Renilla* luciferase activity. The NSP6 expression in the transfected cells at 24 h post-transfection was confirmed by western blotting.

### IFN signaling assay

The IFN signaling assay was performed as previously reported (19) with some modifications. HEK293T cells were seeded onto 24-well plates and cultured overnight at 37°C. The cells were transfected with the indicated viral protein expression plasmids or pCAGGS empty vector (500 ng), the ISRE-driven firefly luciferase-expressing reporter plasmid pISRE-luc (500 ng; Clontech), and pGL4.74 [hRluc/TK] vector (50 ng; Promega), which served as a transfection control. At 16 h post-transfection, the transfected cells were treated with 1,000 units/mL of human IFN-α (Millipore). After an 8 h incubation, the cells were lysed, and firefly and *Renilla* luciferase activities were measured by using the Dual-Glo luciferase assay system (Promega). The NSP6 expression in the transfected cells at 24 h post-transfection was confirmed by western blotting.

### Western blotting

Cells were lysed in Tris-Glycine SDS sample buffer (Invitrogen), sonicated for 5 min, and then subjected to SDS-PAGE on Any kD Mini-PROTEAN TGM Precast Protein Gels (Bio-Rad). Proteins in the SDS-PAGE gels were transferred to a polyvinylidene fluoride membrane (Millipore) and detected by using the indicated primary antibodies (mouse anti-DYKDDDDK tag monoclonal antibody [Clone No.1E6, Wako, 1:5,000] or mouse anti-β-actin [Sigma-Aldrich, 1:10,000]), followed by secondary antibodies (sheep horseradish peroxidase-conjugated anti-mouse IgG [GE Healthcare]). Signals were developed by using ECL Prime Western Blotting Detection Reagent (GE Healthcare). Images were captured with the ChemiDoc Touch Imaging System (Bio-Rad).

### Statistical analysis

GraphPad Prism software was used to analyze all data. Differences among groups were considered significant for *P* values < 0.05.

## Data Availability

All data supporting the findings of this study are available within the paper and from the corresponding author upon request. There are no restrictions to obtaining access to the primary data.

## SUPPLEMENTAL MATERIAL

Figure S1 jvi.00656-25-s0001.tif Uncropped image of Fig. 4.

ASM does not own the copyrights to Supplemental Material that may be linked to, or accessed through, an article. The authors have granted ASM a non-exclusive, world-wide license to publish the Supplemental Material files. Please contact the corresponding author directly for reuse.

## References

[1] Zhu N, Zhang D, Wang W, Li X, Yang B, Song J, Zhao X, Huang B, Shi W, Lu R, Niu P, Zhan F, Ma X, Wang D, Xu W, Wu G, Gao GF, Tan W, China Novel Coronavirus Investigating and Research Team. 2020. A novel coronavirus from patients with pneumonia in China, 2019. N Engl J Med 382:727–733. doi:10.1056/NEJMoa2001017 31978945 PMC7092803

[2] Huang C, Wang Y, Li X, Ren L, Zhao J, Hu Y, Zhang L, Fan G, Xu J, Gu X, et al.. 2020. Clinical features of patients infected with 2019 novel coronavirus in Wuhan, China. The Lancet 395:497–506. doi:10.1016/S0140-6736(20)30183-5 PMC7159299 31986264

[3] Malone B, Urakova N, Snijder EJ, Campbell EA. 2022. Structures and functions of coronavirus replication-transcription complexes and their relevance for SARS-CoV-2 drug design. Nat Rev Mol Cell Biol 23:21–39. doi:10.1038/s41580-021-00432-z 34824452 PMC8613731

[4] Ricciardi S, Guarino AM, Giaquinto L, Polishchuk EV, Santoro M, Di Tullio G, Wilson C, Panariello F, Soares VC, Dias SSG, Santos JC, Souza TML, Fusco G, Viscardi M, Brandi S, Bozza PT, Polishchuk RS, Venditti R, De Matteis MA. 2022. The role of NSP6 in the biogenesis of the SARS-CoV-2 replication organelle. Nature 606:761–768. doi:10.1038/s41586-022-04835-6 35551511 PMC7612910

[5] Bills C, Xie X, Shi P-Y. 2023. The multiple roles of nsp6 in the molecular pathogenesis of SARS-CoV-2. Antiviral Res 213:105590. doi:10.1016/j.antiviral.2023.105590 37003304 PMC10063458

[6] Chen D-Y, Chin CV, Kenney D, Tavares AH, Khan N, Conway HL, Liu G, Choudhary MC, Gertje HP, O’Connell AK, Adams S, Kotton DN, Herrmann A, Ensser A, Connor JH, Bosmann M, Li JZ, Gack MU, Baker SC, Kirchdoerfer RN, Kataria Y, Crossland NA, Douam F, Saeed M. 2023. Spike and nsp6 are key determinants of SARS-CoV-2 Omicron BA.1 attenuation. Nature 615:143–150. doi:10.1038/s41586-023-05697-2 36630998

[7] Taha TY, Chen IP, Hayashi JM, Tabata T, Walcott K, Kimmerly GR, Syed AM, Ciling A, Suryawanshi RK, Martin HS, Bach BH, Tsou C-L, Montano M, Khalid MM, Sreekumar BK, Renuka Kumar G, Wyman S, Doudna JA, Ott M. 2023. Rapid assembly of SARS-CoV-2 genomes reveals attenuation of the Omicron BA.1 variant through NSP6. Nat Commun 14:2308. doi:10.1038/s41467-023-37787-0 37085489 PMC10120482

[8] Uraki R, Halfmann PJ, Iida S, Yamayoshi S, Furusawa Y, Kiso M, Ito M, Iwatsuki-Horimoto K, Mine S, Kuroda M, et al.. 2022. Characterization of SARS-CoV-2 Omicron BA.4 and BA.5 isolates in rodents. Nature 612:540–545. doi:10.1038/s41586-022-05482-7 36323336 PMC12927073

[9] Halfmann PJ, Iwatsuki-Horimoto K, Kuroda M, Hirata Y, Yamayoshi S, Iida S, Uraki R, Ito M, Ueki H, Furusawa Y, Sakai-Tagawa Y, Kiso M, Armbrust T, Spyra S, Maeda K, Wang Z, Imai M, Suzuki T, Kawaoka Y. 2024. Characterization of Omicron BA.4.6, XBB, and BQ.1.1 subvariants in hamsters. Commun Biol 7:331. doi:10.1038/s42003-024-06015-w 38491227 PMC10943235

[10] Imai M, Ito M, Kiso M, Yamayoshi S, Uraki R, Fukushi S, Watanabe S, Suzuki T, Maeda K, Sakai-Tagawa Y, Iwatsuki-Horimoto K, Halfmann PJ, Kawaoka Y. 2023. Efficacy of antiviral agents against Omicron subvariants BQ.1.1 and XBB. N Engl J Med 388:89–91. doi:10.1056/NEJMc2214302 36476720 PMC9749618

[11] Yamasoba D, Uriu K, Plianchaisuk A, Kosugi Y, Pan L, Zahradnik J, Genotype to Phenotype Japan (G2P-Japan) Consortium, Ito J, Sato K. 2023. Virological characteristics of the SARS-CoV-2 Omicron XBB.1.16 variant. Lancet Infect Dis 23:655–656. doi:10.1016/S1473-3099(23)00278-5 37148902 PMC10156138

[12] Karyakarte RP, Das R, Rajmane MV, Dudhate S, Agarasen J, Pillai P, Chandankhede PM, Labhshetwar RS, Gadiyal Y, Kulkarni PP, Nizarudeen S, Joshi S, Karmodiya K, Potdar V. 2023. Chasing SARS-CoV-2 XBB.1.16 recombinant lineage in India and the clinical profile of XBB.1.16 cases in Maharashtra, India. Cureus 15:e39816. doi:10.7759/cureus.39816 37397651 PMC10314318

[13] Imai M, Iwatsuki-Horimoto K, Hatta M, Loeber S, Halfmann PJ, Nakajima N, Watanabe T, Ujie M, Takahashi K, Ito M, et al.. 2020. Syrian hamsters as a small animal model for SARS-CoV-2 infection and countermeasure development. Proc Natl Acad Sci USA 117:16587–16595. doi:10.1073/pnas.2009799117 32571934 PMC7368255

[14] Sia SF, Yan L-M, Chin AWH, Fung K, Choy K-T, Wong AYL, Kaewpreedee P, Perera R, Poon LLM, Nicholls JM, Peiris M, Yen H-L. 2020. Pathogenesis and transmission of SARS-CoV-2 in golden hamsters. Nature 583:834–838. doi:10.1038/s41586-020-2342-5 32408338 PMC7394720

[15] Chan J-W, Zhang AJ, Yuan S, Poon V-M, Chan C-S, Lee A-Y, Chan W-M, Fan Z, Tsoi H-W, Wen L, Liang R, Cao J, Chen Y, Tang K, Luo C, Cai J-P, Kok K-H, Chu H, Chan K-H, Sridhar S, Chen Z, Chen H, To K-W, Yuen K-Y. 2020. Simulation of the clinical and pathological manifestations of coronavirus disease 2019 (COVID-19) in a golden Syrian hamster model: implications for disease pathogenesis and transmissibility. Clin Infect Dis 71:2428–2446. doi:10.1093/cid/ciaa325 32215622 PMC7184405

[16] Knoops K, Kikkert M, Worm SHE van den, Zevenhoven-Dobbe JC, van der Meer Y, Koster AJ, Mommaas AM, Snijder EJ. 2008. SARS-coronavirus replication is supported by a reticulovesicular network of modified endoplasmic reticulum. PLoS Biol 6:e226. doi:10.1371/journal.pbio.0060226 18798692 PMC2535663

[17] Yang H, Lyu Y, Hou F. 2020. SARS-CoV-2 infection and the antiviral innate immune response. J Mol Cell Biol 12:963–967. doi:10.1093/jmcb/mjaa071 33377937 PMC7798998

[18] Ueno S, Amarbayasgalan S, Sugiura Y, Takahashi T, Shimizu K, Nakagawa K, Kawabata-Iwakawa R, Kamitani W. 2024. Eight-amino-acid sequence at the N-terminus of SARS-CoV-2 nsp1 is involved in stabilizing viral genome replication. Virology (Auckl) 595:110068. doi:10.1016/j.virol.2024.110068 38593595

[19] Xia H, Cao Z, Xie X, Zhang X, Chen J-C, Wang H, Menachery VD, Rajsbaum R, Shi P-Y. 2020. Evasion of type I interferon by SARS-CoV-2. Cell Rep 33:108234. doi:10.1016/j.celrep.2020.108234 32979938 PMC7501843

[20] Yamayoshi S, Watanabe M, Goto H, Kawaoka Y. 2016. Identification of a novel viral protein expressed from the PB2 segment of influenza A virus. J Virol 90:444–456. doi:10.1128/JVI.02175-15 26491155 PMC4702538

[21] Yeh T-Y, Contreras GP. 2021. Viral transmission and evolution dynamics of SARS-CoV-2 in shipboard quarantine. Bull World Health Organ 99:486–495. doi:10.2471/BLT.20.255752 34248221 PMC8243027

[22] Al-Khatib HA, Smatti MK, Ali FH, Zedan HT, Thomas S, Ahmed MN, El-Kahlout RA, Al Bader MA, Elgakhlab D, Coyle PV, Abu-Raddad LJ, Al Thani AA, Yassine HM. 2022. Comparative analysis of within-host diversity among vaccinated COVID-19 patients infected with different SARS-CoV-2 variants. iScience 25:105438. doi:10.1016/j.isci.2022.105438 36310647 PMC9595287

[23] Stolp B, Stern M, Ambiel I, Hofmann K, Morath K, Gallucci L, Cortese M, Bartenschlager R, Ruggieri A, Graw F, Rudelius M, Keppler OT, Fackler OT. 2022. SARS-CoV-2 variants of concern display enhanced intrinsic pathogenic properties and expanded organ tropism in mouse models. Cell Rep 38:110387. doi:10.1016/j.celrep.2022.110387 35134331 PMC8795826

[24] Adney DR, Lovaglio J, Schulz JE, Yinda CK, Avanzato VA, Haddock E, Port JR, Holbrook MG, Hanley PW, Saturday G, Scott D, Shaia C, Nelson AM, Spengler JR, Tansey C, Cossaboom CM, Wendling NM, Martens C, Easley J, Yap SW, Seifert SN, Munster VJ. 2022. Severe acute respiratory disease in American mink experimentally infected with SARS-CoV-2. JCI Insight 7:7. doi:10.1172/jci.insight.159573 PMC9746805 36509288

[25] Carlin AF, Clark AE, Chaillon A, Garretson AF, Bray W, Porrachia M, Santos AT, Rana TM, Smith DM. 2023. Virologic and Immunologic characterization of coronavirus disease 2019 recrudescence after nirmatrelvir/ritonavir treatment. Clin Infect Dis 76:e530–e532. doi:10.1093/cid/ciac496 35723411 PMC9278181

[26] Torii S, Kim KS, Koseki J, Suzuki R, Iwanami S, Fujita Y, Jeong YD, Ito J, Asakura H, Nagashima M, Sadamasu K, Yoshimura K, Genotype to Phenotype Japan (G2P-Japan) Consortium, Sato K, Matsuura Y, Shimamura T, Iwami S, Fukuhara T. 2023. Increased flexibility of the SARS-CoV-2 RNA-binding site causes resistance to remdesivir. PLOS Pathog 19:e1011231. doi:10.1371/journal.ppat.1011231 36972312 PMC10089321

[27] Feng Y, Yi J, Yang L, Wang Y, Wen J, Zhao W, Kim P, Zhou X. 2024. COV2Var, a function annotation database of SARS-CoV-2 genetic variation. Nucleic Acids Res 52:D701–D713. doi:10.1093/nar/gkad958 37897356 PMC10767816

[28] Dobson L, Reményi I, Tusnády GE. 2015. CCTOP: a consensus constrained TOPology prediction web server. Nucleic Acids Res 43:W408–12. doi:10.1093/nar/gkv451 25943549 PMC4489262

[29] Benvenuto D, Angeletti S, Giovanetti M, Bianchi M, Pascarella S, Cauda R, Ciccozzi M, Cassone A. 2020. Evolutionary analysis of SARS-CoV-2: how mutation of non-structural protein 6 (NSP6) could affect viral autophagy. J Infect 81:e24–e27. doi:10.1016/j.jinf.2020.03.058 PMC7195303 32283146

[30] Omasits U, Ahrens CH, Müller S, Wollscheid B. 2014. Protter: interactive protein feature visualization and integration with experimental proteomic data. Bioinformatics 30:884–886. doi:10.1093/bioinformatics/btt607 24162465

[31] Kumar A, Kumar P, Saumya KU, Giri R. 2021. Investigating the conformational dynamics of SARS-CoV-2 NSP6 protein with emphasis on non-transmembrane 91-112 & 231-290 regions. Microb Pathog 161:105236. doi:10.1016/j.micpath.2021.105236 34648928 PMC8505028

[32] Pandey P, Prasad K, Prakash A, Kumar V. 2020. Insights into the biased activity of dextromethorphan and haloperidol towards SARS-CoV-2 NSP6: in silico binding mechanistic analysis. J Mol Med (Berl) 98:1659–1673. doi:10.1007/s00109-020-01980-1 32965508 PMC7509052

[33] Taha TY, Ezzatpour S, Hayashi JM, Ye C, Zapatero-Belinchón FJ, Rosecrans JA, Kimmerly GR, Chen IP, Walcott K, Kurianowicz A, et al.. 2025. Enhanced RNA replication and pathogenesis in recent SARS-CoV-2 variants harboring the L260F mutation in NSP6. PLoS Pathog 21:e1013020. doi:10.1371/journal.ppat.1013020 40163530 PMC11981139

[34] So RTY, Chu DKW, Hui KPY, Mok CKP, Shum MHH, Sanyal S, Nicholls JM, Ho JCW, Cheung M-C, Ng K-C, Yeung H-W, Chan MCW, Poon LLM, Zhao J, Lam TTY, Peiris M. 2023. Amino acid substitution L232F in non-structural protein 6 identified as a possible human-adaptive mutation in clade B MERS coronaviruses. J Virol 97:e0136923. doi:10.1128/jvi.01369-23 38038429 PMC10734512

[35] Zhang Y, Xin B, Liu Y, Jiang W, Han W, Deng J, Wang P, Hong X, Yan D. 2023. SARS-COV-2 protein NSP9 promotes cytokine production by targeting TBK1. Front Immunol 14:1211816. doi:10.3389/fimmu.2023.1211816 37854611 PMC10580797

[36] Lundrigan E, Toudic C, Pennock E, Pezacki JP. 2024. SARS-CoV-2 protein Nsp9 is involved in viral evasion through interactions with innate immune pathways. ACS Omega 9:26428–26438. doi:10.1021/acsomega.4c02631 38911767 PMC11191075

[37] Tanner JA, Watt RM, Chai Y-B, Lu L-Y, Lin MC, Peiris JSM, Poon LLM, Kung H-F, Huang J-D. 2003. The severe acute respiratory syndrome (SARS) coronavirus NTPase/helicase belongs to a distinct class of 5’ to 3’ viral helicases. J Biol Chem 278:39578–39582. doi:10.1074/jbc.C300328200 12917423 PMC8060950

[38] Chen J, Malone B, Llewellyn E, Grasso M, Shelton PMM, Olinares PDB, Maruthi K, Eng ET, Vatandaslar H, Chait BT, Kapoor TM, Darst SA, Campbell EA. 2020. Structural basis for helicase-polymerase coupling in the SARS-CoV-2 replication-transcription complex. Cell 182:1560–1573. doi:10.1016/j.cell.2020.07.033 32783916 PMC7386476

[39] Jia Z, Yan L, Ren Z, Wu L, Wang J, Guo J, Zheng L, Ming Z, Zhang L, Lou Z, Rao Z. 2019. Delicate structural coordination of the severe acute respiratory syndrome coronavirus Nsp13 upon ATP hydrolysis. Nucleic Acids Res 47:6538–6550. doi:10.1093/nar/gkz409 31131400 PMC6614802

[40] Robson F, Khan KS, Le TK, Paris C, Demirbag S, Barfuss P, Rocchi P, Ng W-L. 2020. Coronavirus RNA proofreading: molecular basis and therapeutic targeting. Mol Cell 79:710–727. doi:10.1016/j.molcel.2020.07.027 32853546 PMC7402271

[41] Smith EC, Denison MR. 2013. Coronaviruses as DNA wannabes: a new model for the regulation of RNA virus replication fidelity. PLoS Pathog 9:e1003760. doi:10.1371/journal.ppat.1003760 24348241 PMC3857799

[42] Minskaia E, Hertzig T, Gorbalenya AE, Campanacci V, Cambillau C, Canard B, Ziebuhr J. 2006. Discovery of an RNA virus 3’->5’ exoribonuclease that is critically involved in coronavirus RNA synthesis. Proc Natl Acad Sci USA 103:5108–5113. doi:10.1073/pnas.0508200103 16549795 PMC1458802

[43] Moeller NH, Shi K, Demir Ö, Belica C, Banerjee S, Yin L, Durfee C, Amaro RE, Aihara H. 2022. Structure and dynamics of SARS-CoV-2 proofreading exoribonuclease ExoN. Proc Natl Acad Sci USA 119:e2106379119. doi:10.1073/pnas.2106379119 35165203 PMC8892293

[44] Ramaiah A, Khubbar M, Akinyemi K, Bauer A, Carranza F, Weiner J, Bhattacharyya S, Payne D, Balakrishnan N. 2023. Genomic surveillance reveals the rapid expansion of the XBB lineage among circulating SARS-CoV-2 Omicron lineages in Southeastern Wisconsin, USA. Viruses 15:1940. doi:10.3390/v15091940 37766346 PMC10535685

[45] Matsuyama S, Nao N, Shirato K, Kawase M, Saito S, Takayama I, Nagata N, Sekizuka T, Katoh H, Kato F, Sakata M, Tahara M, Kutsuna S, Ohmagari N, Kuroda M, Suzuki T, Kageyama T, Takeda M. 2020. Enhanced isolation of SARS-CoV-2 by TMPRSS2-expressing cells. Proc Natl Acad Sci USA 117:7001–7003. doi:10.1073/pnas.2002589117 32165541 PMC7132130

[46] Imai M, Halfmann PJ, Yamayoshi S, Iwatsuki-Horimoto K, Chiba S, Watanabe T, Nakajima N, Ito M, Kuroda M, Kiso M, et al.. 2021. Characterization of a new SARS-CoV-2 variant that emerged in Brazil. Proc Natl Acad Sci USA 118:e2106535118. doi:10.1073/pnas.2106535118 34140350 PMC8271735

[47] Halfmann PJ, Uraki R, Kuroda M, Iwatsuki-Horimoto K, Yamayoshi S, Ito M, Kawaoka Y. 2023. Transmission and re-infection of Omicron variant XBB.1.5 in hamsters. EBioMedicine 93:104677. doi:10.1016/j.ebiom.2023.104677 37352827 PMC10329098

[48] Uraki R, Ito M, Kiso M, Yamayoshi S, Iwatsuki-Horimoto K, Furusawa Y, Sakai-Tagawa Y, Imai M, Koga M, Yamamoto S, Adachi E, Saito M, Tsutsumi T, Otani A, Kikuchi T, Yotsuyanagi H, Halfmann PJ, Pekosz A, Kawaoka Y. 2023. Antiviral and bivalent vaccine efficacy against an Omicron XBB.1.5 isolate. Lancet Infect Dis 23:402–403. doi:10.1016/S1473-3099(23)00070-1 36773622 PMC9908083

[49] Uraki R, Kiso M, Iwatsuki-Horimoto K, Yamayoshi S, Ito M, Chiba S, Sakai-Tagawa Y, Imai M, Kashima Y, Koga M, Fuwa N, Okumura N, Hojo M, Iwamoto N, Kato H, Nakajima H, Ohmagari N, Yotsuyanagi H, Suzuki Y, Kawaoka Y. 2023. Characterization of a SARS-CoV-2 EG.5.1 clinical isolate in vitro and in vivo. Cell Rep 42:113580. doi:10.1016/j.celrep.2023.113580 38103202 PMC12927040

[50] Uraki R, Ito M, Furusawa Y, Yamayoshi S, Iwatsuki-Horimoto K, Adachi E, Saito M, Koga M, Tsutsumi T, Yamamoto S, Otani A, Kiso M, Sakai-Tagawa Y, Ueki H, Yotsuyanagi H, Imai M, Kawaoka Y. 2023. Humoral immune evasion of the Omicron subvariants BQ.1.1 and XBB. Lancet Infect Dis 23:30–32. doi:10.1016/S1473-3099(22)00816-7 36495917 PMC9729000

[51] Rabe BA, Cepko C. 2020. A simple enhancement for gibson isothermal assembly. Molecular Biology. doi:10.1101/2020.06.14.150979

[52] Itokawa K, Sekizuka T, Hashino M, Tanaka R, Kuroda M. 2020. Disentangling primer interactions improves SARS-CoV-2 genome sequencing by multiplex tiling PCR. PLOS ONE 15:e0239403. doi:10.1371/journal.pone.0239403 32946527 PMC7500614

[53] Schröder S, Richter A, Veith T, Emanuel J, Gudermann L, Friedmann K, Jeworowski LM, Mühlemann B, Jones TC, Müller MA, Corman VM, Drosten C. 2023. Characterization of intrinsic and effective fitness changes caused by temporarily fixed mutations in the SARS-CoV-2 spike E484 epitope and identification of an epistatic precondition for the evolution of E484A in variant Omicron. Virol J 20:257. doi:10.1186/s12985-023-02154-4 37940989 PMC10633978

[54] Livak KJ, Schmittgen TD. 2001. Analysis of relative gene expression data using real-time quantitative PCR and the 2(-Delta Delta C(T)) Method. Methods 25:402–408. doi:10.1006/meth.2001.1262 11846609

[55] Shuai H, Chan JF-W, Hu B, Chai Y, Yuen TT-T, Yin F, Huang X, Yoon C, Hu J-C, Liu H, et al.. 2022. Attenuated replication and pathogenicity of SARS-CoV-2 B.1.1.529 Omicron. Nature 603:693–699. doi:10.1038/s41586-022-04442-5 35062016

[56] Dagotto G, Mercado NB, Martinez DR, Hou YJ, Nkolola JP, Carnahan RH, Crowe JE Jr, Baric RS, Barouch DH. 2021. Comparison of subgenomic and total RNA in SARS-CoV-2 challenged rhesus macaques. J Virol 95:e02370-20. doi:10.1128/JVI.02370-20 33472939 PMC8103707

[57] Gunalan V, Mirazimi A, Tan Y-J. 2011. A putative diacidic motif in the SARS-CoV ORF6 protein influences its subcellular localization and suppression of expression of co-transfected expression constructs. BMC Res Notes 4:446. doi:10.1186/1756-0500-4-446 22026976 PMC3219739

[58] Yoneyama M, Suhara W, Fukuhara Y, Fukuda M, Nishida E, Fujita T. 1998. Direct triggering of the type I interferon system by virus infection: activation of a transcription factor complex containing IRF-3 and CBP/p300. EMBO J 17:1087–1095. doi:10.1093/emboj/17.4.1087 9463386 PMC1170457

