# Supplementary figures and images for: The NSP6-L260F substitution in SARS-CoV-2 BQ.1.1 and XBB.1.16 lineages compensates for the reduced viral polymerase activity caused by mutations in NSP13 and NSP14
Source: J Virol. 2025 May 13;99(6):e00656-25. doi: 10.1128/jvi.00656-25 (PMC12172475; doi:10.1128/jvi.00656-25)

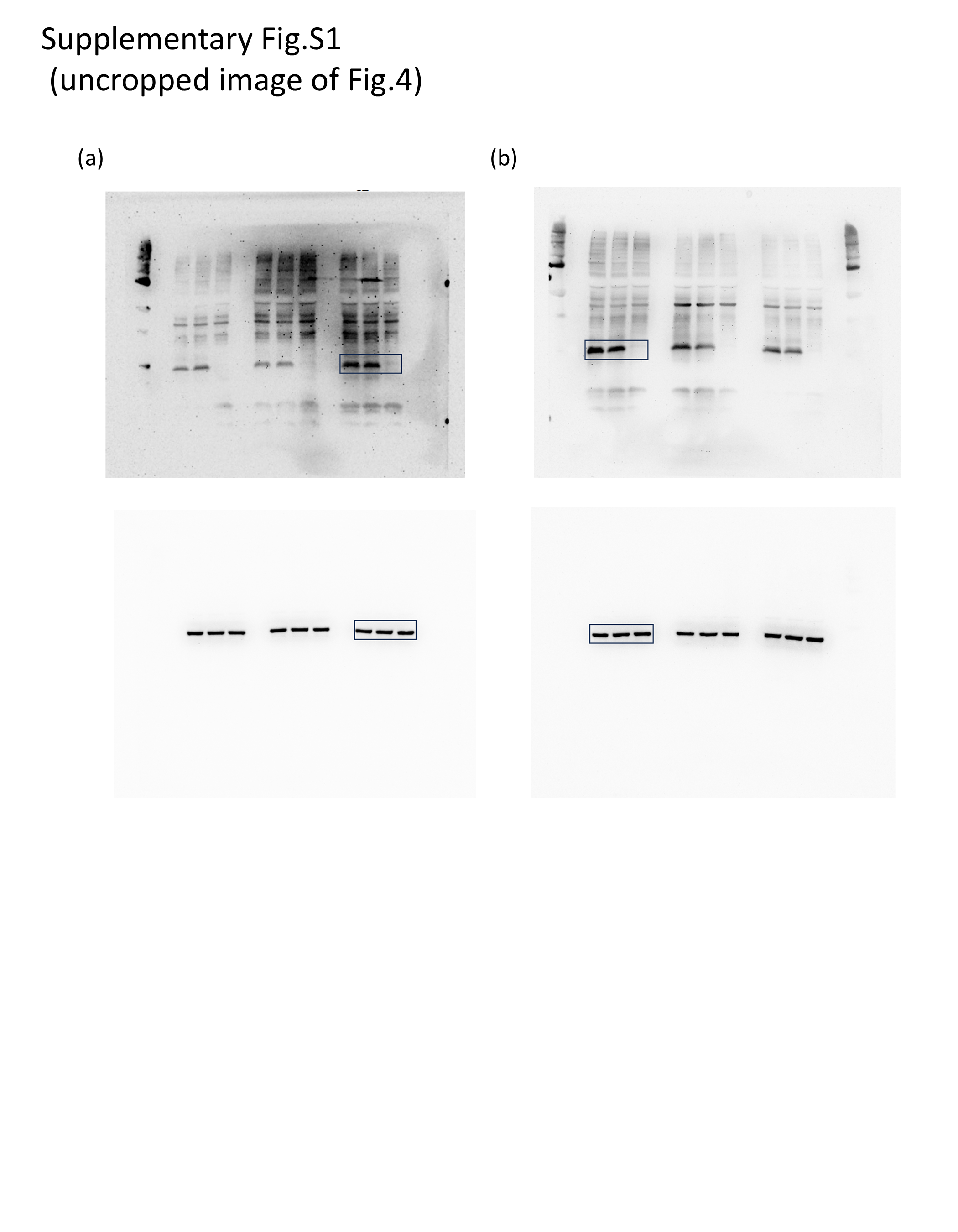

Supplement: Figure S1 — Uncropped image of Fig. 4. [file jvi.00656-25-s0001.tif]
